# Assessment of Gastroenteric Viruses Frequency in a Children's Day Care Center in Rio De Janeiro, Brazil: A Fifteen Year Study (1994–2008)

**DOI:** 10.1371/journal.pone.0033754

**Published:** 2012-03-20

**Authors:** Mônica Simões Rocha Ferreira, Maria da Penha Trindade Pinheiro Xavier, Anna Carolina De Castro Tinga, Tatiana Lundgren Rose, Tulio Machado Fumian, Alexandre Madi Fialho, Rosane Maria de Assis, Filipe Aníbal Carvalho Costa, Solange Artimos de Oliveira, José Paulo Gagliardi Leite, Marize Pereira Miagostovich

**Affiliations:** 1 Laboratório de Virologia Comparada e Ambiental, Instituto Oswaldo Cruz/Fiocruz, Rio de Janeiro, Rio de Janeiro, Brazil; 2 Laboratório de Sistemática Bioquímica, Instituto Oswaldo Cruz/Fiocruz, Rio de Janeiro, Rio de Janeiro, Brazil; 3 Disciplina de Doenças Infecciosas e Parasitárias, Faculdade de Medicina, Universidade Federal Fluminense, Niterói, Rio de Janeiro, Brazil; Duke University School of Medicine, United States of America

## Abstract

This 15-year study aimed to determine the role of the main viruses responsible for acute infantile gastroenteritis cases in a day care center in the city of Rio de Janeiro, Brazil. From 1994 to 2008, 539 fecal samples were obtained from 23 outbreaks as well as sporadic cases that occurred in this period. The detection of Rotavirus group A (RVA), norovirus (NoV) and astrovirus (AstV) was investigated both by classical and molecular methods of viral detection. RVA was detected by enzymatic immune assay and/or polyacrylamide gel electrophoresis and genotyped by using semi-nested multiplex PCR. NoV and AstV were subsequently tested by real time PCR in all RVA-negative samples and genotyped throughout genome sequencing. Three protocols for molecular characterization of NoV nucleotide sequencing were performed with the partial nucleotide sequencing of genomic regions known as region B (polymerase gen), C and D (capsid gen).Viruses were identified in 47.7% (257/539) of the cases, and the detection rates of RVA, NoV and AstV in16.1% (87/539), 33.4% (151/452), and 6.3% (19/301), respectively. Most gastroenteritis cases were reported in autumn and winter, although NoV presented a broader monthly distribution. Viruses' detection rates were significantly higher among children aged less than 24 months old, although NoV cases were detected in all age groups. RVA genotypes as G1P[8], G9P[8], G2P[4], G3P[8] and G1+G3P[8] and RVA was no longer detected after 2005. NoV characterization revealed genotypes variability circulating in the period as GI.2, GI.3, GI.8 GII.2, GII.3, GII.4, GII.4 variants 2001 and 2006b, GII.6, GII.7, GII.12 and GII.17. AstV genotypes 1, 2, 4 and 5 were also characterized. Those data demonstrate the impact of NoV infection in cases of infantile gastroenteritis, surpassing RVA infection responsible for high morbidity rate in children under five years old.

## Introduction

Acute gastroenteritis (AGE) is an important health concern and can be caused by a wide array of viruses many of which identified in the 70's [1], [2], [3]. Approximately 2.0 million deaths related to diarrhea are reported each year, occurring mainly in low as well as in middle income countries presenting no defined etiological diagnosis [4]. Globally, rotavirus group A (RVA) is responsible for around 440 000 deaths each year among children under 5 years old known worldwide as the most important viral agent in severe cases of AGE [5].

Following the World Health Organization recommendations, a monovalent human rotavirus vaccine (Rotarix®; Glaxo Smith Kline Biologicals, Rixensart, Belgium) was included in 2006 in the Brazilian National Immunization Program as well as in other countries in the Americas and in Europe [4], [6]. Recently, a meta-analysis study evaluating the effects of RVA vaccination showed that the impact of the global burden of severe diarrhea in childhood vaccination varies according to its geographic region [7].

After RVA, norovirus (NoV) and astrovirus (AstV) are the most important etiologic agents of infantile AGE worldwide [8], [9], [10], [11], [12] associated with sporadic cases and outbreaks in indoor settings such as schools, cruise chips, restaurants, hospitals, homes for the elderly and military recruits [13]. Incidence rates of those viruses among children aged less than 5 years-old, represent a particular concern in infants attending nurseries [14], [15], [16].

Currently, day care facilities are a very common type of service offered in many regions throughout the world, as part of everyday life for many families living in urban areas, however it has been observed that children attending those centers are at increased risk of acquiring gastroenteritis [15], [17], [18,].

In Brazil, there are few studies reporting the burden of viral gastroenteritis and its etiological profile in this population [19], [20], [21], [22]. Moreover, no studies regarding the detection rates of the three main viral agents associated with AGE and discriminating sporadic cases and outbreaks on a regular basis have been reported yet. This study aims to assess the impact of those viruses in cases of infantile AGE that occurred in a day care center in the city of Rio de Janeiro, Brazil, between 1994 and 2008. The broad scope of this study (15 years) allowed the evaluation of different epidemiological aspects of these viruses including seasonality, genetic diversity, and preventive measures as well as aspects related to Rotarix® vaccine introduced in Brazil in 2006.

## Materials and Methods

### Case definition

AGE cases were defined by the presence of diarrhoea (three or more loose or liquid bowel movements per day) accompanied or not by fever and/or vomiting. This definition was used for sporadic or outbreak cases. An outbreak was defined as the occurrence of an AGE case detected in three or more infants within one week. The occurrence of other AGE cases in the following week was considered as one outbreak.

### Setting, collection of faecal samples and study design

Bertha Lutz day care center is located at the *campus* of the Oswaldo Cruz Foundation, Rio de Janeiro, Brazil and caters for children ranging from four months of age, who can remains at an average of eight hours per day in periods that can last up to five years. Given the number of hours that the children are collectively indoors they receive proper health care that provides the services of a paediatrician, a nurse and a health technician.

As an institutional facility, this day care serves its employees who live in different municipalities and that belong to distinct socio economic levels. The nursery serves about 200 children that are organized in classes according to their age group such as: pre-nursery (four months of age up to one year and four months old), nursery (one year and five months old up to two years and three months old); maternal (two years and four months old up to three years and 11 months of age) and kindergarten (four years old up to five years of age).

Parasitological researches including *Giardia sp*. are periodically conducted in children (every 6 months) and in the day care professional staff (annually), a pre requisite for enrolments. When a case of AGE is reported, stool samples are collected by medical staff and sent to the institutional laboratories to clarify the specific etiologic agent. Bacterial investigation usually includes *Campylobacter sp.*, *Shigella sp. and Salmonela sp*.

AGE cases from outbreaks or sporadic cases reported in the day care center between 1994 and 2008 previously negative for these agents were included in this study. All specimens were obtained during the acute phase of illness (i.e., within 24–72 hours after onset of symptoms). Samples taken from children were collected by medical staff and in some cases, parents were instructed to collect samples at home, as well as the employees affected. Stool samples from each individual were collected in plastic bottles and preserved at 4°C until initial processing.

### Ethics

Ethical approval for this study was obtained from Fundação Oswaldo Cruz Ethics Committee (CEP 311/06). A written informed consent was obtained from the patient's parents or legal guardians for all samples collected.

### Viruses' detection and genotyping

Twenty per cent (1 g/5 mL) of stool suspensions were prepared in 0.01 M Tris-HCl 0.0015 M Ca^2+^ (pH 7.2). For RVA, nucleic acid extraction was performed with glass-powder method modified [23], [24]. The QIAamp Viral RNA™ extraction kit (QIAGEN®, Valencia, CA, USA) was performed for NoV and AstV extraction.

Table 1 presents the methodologies used for viruses' detection and characterization including references. Screening for RVA was performed in all samples by the enzyme immunoassays (EIA) and by detection using the polyacrylamide gel electrophoresis (PAGE) method [25]. RVA positive samples were genotyped using semi-nested multiplex PCR for VP4 and VP7 gen amplification. NoV was tested in RVA-negative samples. Subsequently, all RVA and NoV negative samples were analyzed for AstV. The detection of NoV and AstV was performed by real time PCR. Specific primers and probes for TaqMan® system (Applied Biosystems, Foster City, USA) were used and data were collected and analyzed using the Sequence Detector software version 1.6 (Applied Biosystems). [26]a [27]a [28]a [29]a [30]s [31]d [32]a [33]s For NoV and AstV genotyping different PCR protocols were used for nucleotide sequencing products (Table 1). Amplicons obtained from Region B, C or D of NoV and partial ORF2 region for AstV were purified using the QIAquick® PCR Purification Kit (QIAGEN, CA, USA) following manufacturer's recommendations. The amplicons were analyzed by 2% agarose gel electrophoresis using the Low DNA Mass Ladder (Invitrogen®, CA, USA) for the recognition of molecular patterns. PCR products were sequenced in both directions using an ABI Prism 3100 Genetic Analyzer and Big Dye Terminator Cycle Sequencing Kit v. 3.1 (Applied Biosystems, CA, USA as described by [34]. Centri-Sep columns (Princeton Separations, CA, USA) were used for purification of sequencing reaction products according to manufacturer's recommendations. The sequences were aligned and edited using the BioEdit Sequence Alignment Editor [35] and compared with sequences available including genotype reference sequences. A phylogenetic tree was constructed with the MEGA 4 program using the neighbour-joining method, and the genetic distance was calculated using the Kimura 2 parameters model with 2,000 pseudo-replicas for genotypic strain classification [36].

**Table 1:** Methods used for detection and molecular characterization of the samples from cases of of acutegastroenteritis in a day-care center, Rio de Janeiro, Brazil, 1994–2008.

Viruses	Virusesdetection		Virusescharacterization	
	Methods	References	Methods/region	References
**RVA**	PAGE, EIA	[25], IDEA® Rotavirus *or RIDASCREEN® Rotavirus **	Semi-nested multiplex PCR, VP4 (G)/VP7(P)	[26] [27] [24]
**NoV**	Real time PCR	[28]	Region B(RpRd), Region C (Capsid),Region D (Capsid)	[29] [30] [31]
**AstV**	Real time PCR	[32]	ORF2	[33]

* OXOID Ltd, England.

** R Biopharm Group, Germany.

### Positive and negative control

The strains RVA SA11, AstV type 2, which was isolated in cell cultures, NoV GI.3 (access number Genbank: GU132470) and NoV GII.4 (access number GenBank: GU132457) and Milli-Q water were used as positive and negative controls, respectively, in the RNA extraction all through to genomic amplification stage in order to avoid false positive results. The molecular procedures were conducted in four separate rooms to avoid cross contamination of samples. In all procedures, recommended manipulations for PCR techniques were strictly followed as a precaution to avoid false-positive results.

### Statistical analysis

Statistical analyses were performed with EpiInfo 2000 version 3.5.1 (Center for Disease Control and Prevention, Atlanta, Georgia, USA) and SPSS version 15.0 (SPSS Inc, Chicago, Illinois, USA) software. Data were presented as descriptive statistics and frequencies of different virus detection in distinct groups were compared by using the chi-square test, at the 0.05 level of significance. We also calculated odds ratios and their respective 95% confidence intervals.

## Results

RVA, NoV and AstV were detected in 47.7% (257/539) of faecal samples obtained from AGE cases. NoV was the most frequently detected (33.4% [151/452]), followed by RVA (16.1% [87/539]) and AstV (6.3% [19/301]). Taq Man Real time PCR for NoV GI, GII and AstV was performed as rapid assay for viruses' detection and quantification. Standard curves with 10-fold serial dilutions of plasmids containing their targets (from 10^7^ to 10^1^) were generated. A detection limit of 21 (NoV GI) and 10 (NoV GII and AstV) genomic copies per reaction was observed for a threshold cycle (Ct) value not superior to 40.

Table 2 shows the results of virus detection by age group, season, temporal distribution as well as the origin of samples according to sporadic or outbreak cases. Infants aged less than 24 months presented significant higher detection rates of NoV, RVA and AstV, although NoV was frequently detected in all age groups including adults.

**Table 2:** Viral etiology of acute gastroenteritis in a day-care center, Rio de Janeiro, Brazil, 1994–2008.

Variables	RotavirusPositive/examined(%)	NorovirusPositive/examined(%)	AstrovirusPositive/examined(%)	Any viral etiologyPositive/examined(%)
**Age group**				
4–24 months	77/384 (20.1)^a^	114/307 (37.1)^b^	16/193 (8.3)	207/384 (53.9)
25–60 months	7/119 (5.9)^a^	23/112 (20.5)^b^	3/89 (3.4)	33/119 (26.9)
Adults	1/30 (3.3)	12/29 (41.4)	0/17 (0)	13/30 (43.3)
Unknow	2/6	2/4	0/2	4/6
**Season**				
Summer	0/24 (0)	11/24 (45.8)	0/13(0)	11/24 (45.8)
Autumn	40/239 (16.7)	62/199 (31.2)	11/137 (8)	113/239 (47.2)
Winter	31/220 (14.1)	66/189 (34.9)	6/123 (4.9)	103/220 (46.8)
Spring	16/56 (28.6)	12/40 (30)	2/28 (7.1)	30/56 (53.6)
**Temporal distribution**				
1994–2004	87/475 (18.3)	115/388 (29.6)	19/273 (7)	221/475 (46.5)
2005–2008	0/64 (0)	36/64 (56.3)	0/28 (0)	36/64 (56.2)
**Origen of Samples**				
Outbreaks	70/392 (17.9)	107/322 (33.2)	12/215 (5.6)	189/392 (48.1)
Sporadic	17/147 (11.6)	44/130 (28.8)	7/86 (8.1)	68/147 (46.3)
**Total**	87/539 (16.1)	151/452 (33.4)	19/301 (6.3)	257/539 (47.7)

a Odds ratio (OR) = 4.01; 95% confidence interval (CI) = 1.72–9.81; p<0.001.

b OR = 2.29; 95% CI = 1.33–3.95; p = 0.001.

**Figure 1:**
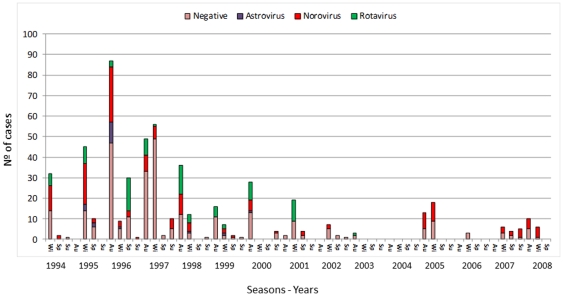
Monthly distribution of viral gastroenteritis outbreaks characterized by 3 or more cases, Rio de Janeiro, Brazil, 1994–2008.

Eighty-five percent of AGE cases (459/539) were reported throughout the autumn and winter seasons, when 84.0% (216/254) of virus-positive cases were observed. NoV presented a broader temporal distribution, detected from summer to spring. AstV and RVA were no longer detected after 2000 and 2003, respectively. The annual distribution of the number of cases (Figure 1) showed a sharp decline from 1994/2000 (average of 61.8 cases/year) to 2001/2008 (13.6 cases/year), when strict hygiene measures were implemented.

**Figure 2:**
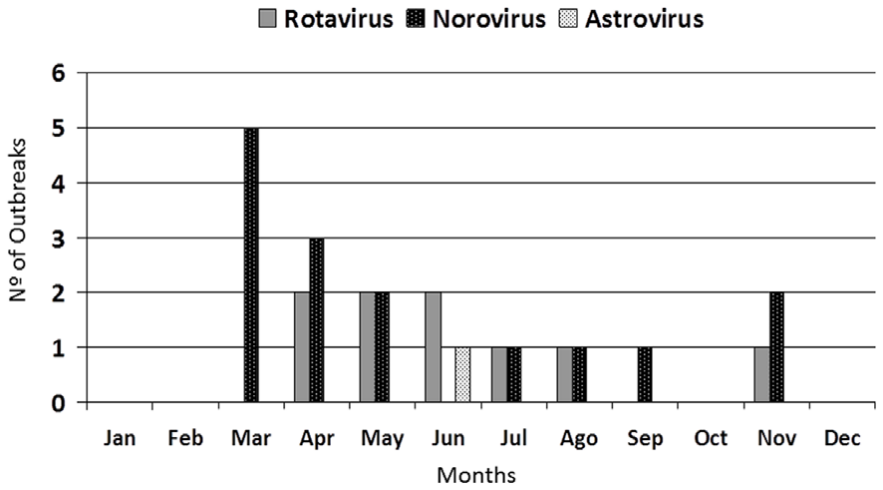
Etiology of viral gastroenteritis in a day-care center by season, Rio de Janeiro, Brazil, 1994–2008 (Wi = Winter, Sp = Spring, Su = Summer, Au = Autumn).

A total of 392 cases of AGE were reported from 23 outbreaks, 18 of them (78.3%) were observed between 1994 and 2000. In 9 outbreaks RVA and NoV were detected circulating simultaneously. In only one outbreak it was observed the co-circulation of the three investigated viruses. Figure 2 shows the monthly cumulative distribution of outbreaks caused by NoV, RVA and AstV in the 15 surveyed years.

**Figure 3:**
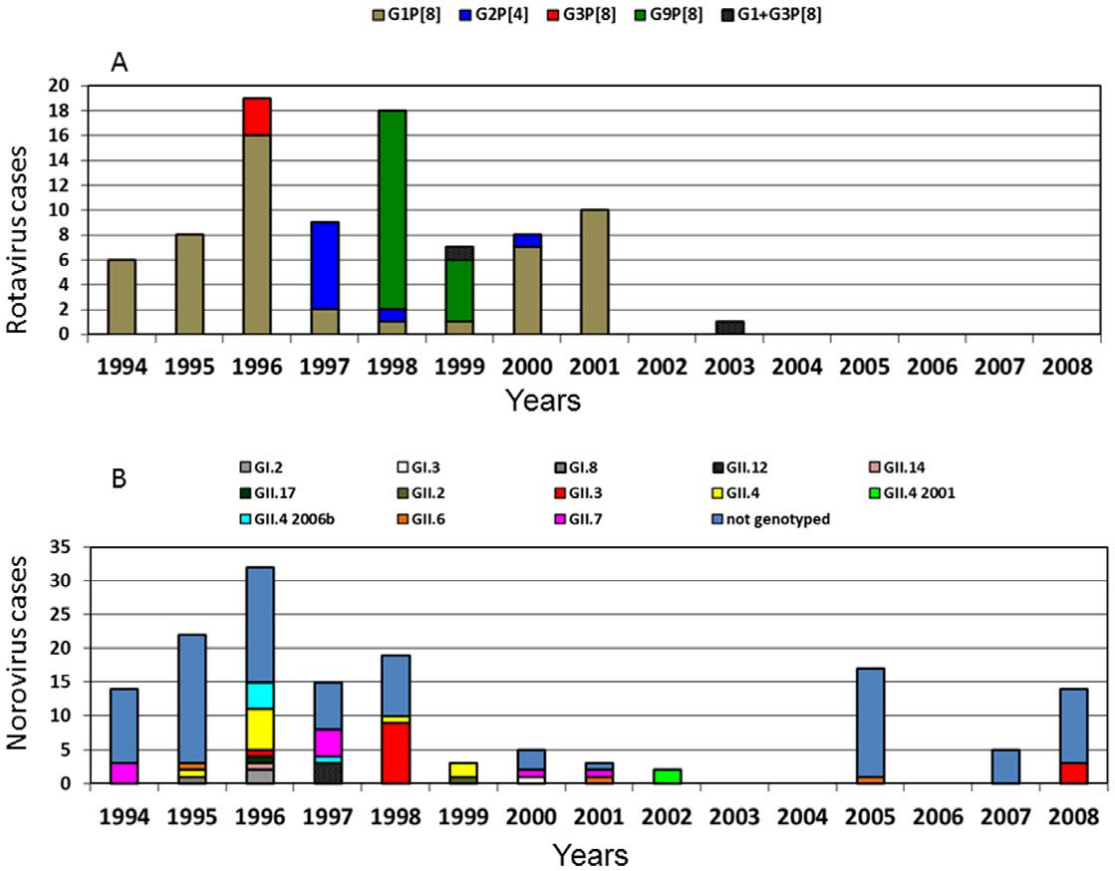
Rotavirus A genotypes (A) and norovirus genotype (B) in a day-care center by year, Rio de Janeiro, Brazil, 1994–2008.

Genotyping of viruses showed a great genetic variability within each group. Figure 3 shows annual distribution of RVA and NoV genotypes from 1994 to 2008. All RVA positive samples were genotyped as G1P[8], G9P[8], G2P[4], G3P[8] or G1+G3P[8]. For NoV genotyping 52 strains were selected to represent outbreaks and sporadic cases distributed over the years. Partial nucleotide sequencing for both GI and GII region D was performed, 31 of them were well characterized. Negative samples for those protocols were then characterized by their region C (9) or B (12) nucleotide sequencing protocols. NoV genotypes were characterized as GI.2, GI.3, GI.8, GII.2, GII.3, GII.4, GII.4 variants 2001 and 2006b, GII.6, GII.7, GII.12 and GII.17. Seven out of 19 AstV strains were well characterized as type 1 (4 strains/1995), 2 (1996), 4 (1998) and 5 (1996).

Nucleotide sequences obtained in this study were submitted to the National Center for Biotechnology Information (GenBank, http://www.ncbi.nlm.nih.gov/) and received the following accession numbers. NoV: 83/1994-JN600530; 270/1995-JN600536; 340/1996-JN600537; 343/1996-JN600531; 345/1996-JN600532; 363/1996-JN600538; 376/1996-JN600535; 407/1996-JN600539; 469/1996-JN600540; 488/1996-JN600541; 489/1996-JN600542; 490/1996-JN600543; 500/1996-JN600544; 505/1996-JN600545; 510/1996-JN600546; 986/1997-JN600547; 1279//1997-JN600548; 1290/1997-JN600549; 1341/1997-JN600550; 512/1998-JN600551; 1515/1998-JN600552; 1518/1998-JN600553; 1532/1998-JN600554; 536/1998JN600555; 1543/1998-JN600556; 1551/1998-JN600557; 1931/1998-JN600558; 2539/1999-JN600559; 2558/1999-JN600560; 2701/1999-JN600561; 3323/2000-JN600562; 4464/2001-JN600563; 5311/2002-JN600564; 5335/2002-JN600565; 10919/2005-JN600567; 15081/2008-JN600566; 15085/2008-JN600533; 15087/2008-JN600534 and AstV: HAstV-251/1995-JQ260759; HAstV-252/1995-JQ260760; HAstV-461/1996-JQ260761; HAstV-473/1996-JQ260762; HAstV-486/1996 -JQ260763; HAstV-1959/1998-JQ260764.

## Discussion

This 15-year study was carried out to assess the frequency of RVA, NoV and AstV as etiologic agents of AGE in children attending a day care center, in Rio de Janeiro, Brazil, since those viruses have been described as the most important viral agents of infantile diarrhea [5], [13], [37]. In low and in middle income countries the detection and molecular characterization of viruses associated with AGE is usually restricted to research and/or reference laboratories and the actual incidence rates described in those countries can, therefore, be underestimated, except for RVA diagnosis that is performed somewhat more regularly due to its low cost.

Among the enteric viruses RVA remain the leading cause of diarrhea in young children in the world [4] with a well documented high morbidity rate in developing countries [5], [6], [7]. However, in this study RVA was the second most detected virus overcome by NoV, whose impact on infant AGE is increasing since the 90 s [11], [13]. In a lowest percentage AstV were also detected. The AstV detection rate found here is in agreement with other studies that demonstrated frequency ranging from 2% to 11% [9], [19], [37], [38], [39].

In this study it was possible to demonstrate the importance of implementing preventive measures to reduce the impact of AGE cases. The joint work performed between laboratory, day care medical and nursing staff can explain the sharp decline on AGE cases from year 2000. Prophylactic measures such as improvement of surface disinfection as well as setting standards for hand washing contributed to reduce incidence rates of AGE cases reported in Bertha Lutz day care center. Hygiene measures taken by adults who work with children have been described as an important factor in reducing cases of viral infection [15], [16], [40]. Medical recommendations of the day care center to keep sick children at home was another of the measurements that has helped reducing the number of cases however it may have also reduced the number of samples collected, since the parents resorted to other medical centers to care and diagnose infection.

Additionally, the authors consider that anti-RVA vaccination may have contributed to the lower detection of RVA comparing to NoV infections. The impact of the immunization of a safe and efficacious oral vaccine G1P [8] in the entire birth cohort in Brazil has been demonstrated both in laboratory and hospital based surveillance studies [41], [42].

In the past, three studies reporting RVA, NoV and AstV outbreaks were carried out at the same institution where the present study was conducted [19]–[22]. Regarding those previous studies it is important to note that RVA outbreak occurred in 1994 [20], before the studied period and that the other outbreaks of NoV [21] and of AstV [19] were already published elsewhere, samples were therefore not included in this study.

Even with a decrease in AGE cases from 2000, the occurrence of NoV outbreaks in subsequent years could explain that NoV infection was near twice as more frequent than infection by RVA corroborating the role of those viruses in childhood infections transmitted indoors [15], [16], [38], [43]. The low NoV infectious dose, the aerosol transmission by vomit and its environmental stability are pointed out as the main characteristics of higher transmissibility of NoV in those specific restricted areas [44], [45]. The susceptibility of adults to NoV infections due to temporary immunity [46] can also be considered as facilitating a factor for transmission, since those professionals either asymptomatic or not could also represent a source of contamination. A highest incidence rate of those infections in pre-nursery and nursery children aged less than two years old corroborated other studies although NoV have been described infecting all age groups [9], [39], [43], [47], [48].

The main limitation of this study in assessing viruses' seasonality lies on the fact that during the summer season the population of the day care center may be substantially reduced due to children's and parents' vacations and that in January the day care center is closed for the holiday season. Nevertheless, a trend towards a higher incidence rate for viral AGE during winter and autumn seasons could be noted as previously observed in Rio de Janeiro, Brazil [41], [49], [50].

Regarding the prevalence of major viral genotypes circulating in this 15 year study it was observed that RVA genotypes corroborated the trends observed in Brazil, i.e. the predominance of G1P[8] from 1994 to 1997 and the emergence of G9P[8] in 1998. Although genotype G2P[4] re-emerged in Brazil from 2005 onwards, the circulation of this genotype in the day care center after 2000 was not detected [22], [41], [50], [51]. The AstV genetic diversity also follows the frequency observed elsewhere being AstV-1 the predominant type, whereas types 2–5 show specific spatial and temporal distribution and types 6–8 are sporadic [52], [53].

For Nov a great genotype diversity was observed since 1996/1997, however the detection of NoV GII.12 and GII.17 prior to its first detection in Brazil [54] have drawn attention revealing the importance of surveillance studies of NoV mainly due to the speed which these viruses spread worldwide. The GII.12 has been described in Japan since 2001 as a major genotype involved in outbreaks [55] and in the U.S. during the winter season 2009/2010, it was responsible for 16% of all reported outbreaks [56]. In Vietnam GII.12 has surpassed in number the cases of GII.4 gastroenteritis in hospitalized children [57]. The same finding occurred in relation to GII.4 2006b variant that was described in different countries causing at least 45 outbreaks on cruise ships throughout Europe in 2006 [58]. Here, this variant was characterized in 1996 responsible for a large outbreak and corroborating the potential of NoV GII.4 variants causing outbreaks around the world [58], [59], [60]. The circulation of GII.4 in the same day care center was demonstrated earlier [21]. The circulation of several NoV strains as GII.3, GII.4, GII.6 and GII.7 in AGE outbreaks in day care centers has been described in different countries [15], [16], [21].

The first description of NoV GI.8 associate to a sporadic case in 1995 also highlighted the relevance of laboratorial surveillance on AGE cases. NoV GI is circulating in a lower prevalence when compared to GII, not only in Brazil but in the world [48], [61], [62], [63], [64], [65].

AGE outbreaks characterized by the co-circulation of more than one virus in this study corroborates the concept that gastrointestinal viruses co-circulate in the same period stressing the need of vigorous control measures during AGE outbreaks affecting people indoors in specific seasons. Unfortunately, the strategy of searching co-infection was not performed. Mixed outbreaks have been described in other day care centers [14], [15], [16] and co-infections especially involving RVA and NoV have also been described [38], [66]. Another limiting factor to assess the real burden of those viruses was not investigating virus excretion in asymptomatic cases. NoV has been detected in those cases and more recently it has been suggested that quantitative analysis should be used to aid clinical interpretation and diagnose when NoV are detected in AGE cases [62], [67].

Concluding, NoV was the main etiological agent found in the studied population, where about half of AGE cases are caused by viruses. These data confirms the role of NoV in indoors outbreaks and pointed out the need to establish laboratory network surveillance that is able to identify the entrance of new viruses or variants in order to prevent outbreaks as well as to provide database for molecular epidemiology studies.

## References

[1] Appleton H, Higgins PG (1975). Viruses and gastroenteritis in infants.. Lancet.

[2] Kapikian AZ, Wyatt RG, Dolin R, Thornhill TS, Kalica AR (1972). Visualization by immune electron microscopy of a 27-nm particle associated with acute infectious nonbacterial gastroenteritis.. J Virol.

[3] Bishop RF, Davidson GP, Holmes IH, Ruck BJ (1973). Virus particles in epithelial cells of duodenal mucosa from children with acute non-bacterial gastroenteritis.. Lancet.

[4] WHO (2009). Initiative for vaccine research (IVR)..

[5] Parashar UD, Gibson CJ, Bresse JS, Glass RI (2006). Rotavirus and severe childhood diarrhea.. Emerg Infect Dis.

[6] Leite JPG, Carvalho-Costa FA, Linhares AC (2008). Group A rotavirus genotypes and the ongoing Brazilian experience: a review.. Mem Inst Oswaldo Cruz.

[7] Walker CF, Black R (2011). Rotavirus vaccine and diarrhea mortality: quantifying regional variation in effect size.. BMC Public Health.

[8] Bereciartu A, Bok K, Gomez J (2002). Identification of viral agents causing gastroenteritis among children in Buenos Aires, Argentina.. Journal of Clinical Virology.

[9] Cardoso DPD, Fiaccadori FS, Borges MDS, Bringel RMM, Leite JPG (2002). Detection and genotyping of astroviruses from children with acute gastroenteritis from Goiania, Goias, Brazil.. Med Sci Monit.

[10] Hansman GS, Doan LTP, Kguyen TA, Okitsu S, Katayama K (2004). Detection of norovirus and sapovirus infection among children with gastroenteritis in Ho Chi Minh City, Vietnam.. Archives of Virology.

[11] Parashar UD, Li J-F, Cama R, De Zalia M, Monroe SS (2004). Human Caliciviruses as a Cause of Severe Gastroenteritis in Peruvian Children.. J Infect Dis.

[12] Gabbay YB, Luz CRN, Costa IV, Cavalcante-Pepino EL, Sousa MS (2005). Prevalence and genetic diversity of astroviruses in children with and without diarrhea in São Luís, Maranhão, Brazil.. Mem Inst Oswaldo Cruz.

[13] Patel MM, Widdowson MA, Glass RI, Akazawa K, Vinje J (2008). Systematic literature review of role of noroviruses in sporadic gastroenteritis.. Emerg Infect Dis.

[14] Taylor MB, Marx FE, Grabow WO (1997). Rotavirus, astrovirus and adenovirus associated with an outbreak of gastroenteritis in a South African child care center.. Epidemiol Infect.

[15] Akihara S, Phan TG, Nguyen TA, Hansman G, Okitsu S (2005). Existence of multiple outbreaks of viral gastroenteritis among infants in a day care center in Japan.. Archives of Virology.

[16] Lyman WH, Walsh JF, Kotch JB, Weber DJ, Gunn E (2009). Prospective Study of Etiologic Agents of Acute Gastroenteritis Outbreaks in Child Care Centers.. J Pediatr.

[17] Hillis SD, Miranda CM, McCann M, Bender D, Weigle K (1992). Day Care Center Attendance and Diarrheal Morbidity in Colombia.. Pediatrics.

[18] Bartlett AV, Moore M, Gary GW, Starko KM, Erben JJ (1985). Diarrheal illness among infants and toddlers in day care centers. I. Epidemiology and pathogens.. J Pediatr.

[19] Silva AMV, Leite EG, Assis RMS, Majerowicz S, Leite JPG (2001). An outbreak of gastroenteritis associated with astrovirus serotype 1 in a day care center, in Rio de Janeiro, Brazil.. Mem Inst Oswaldo Cruz.

[20] Castro L, Rodrigues DP, Flauzino R, Moura M, Leite JPG (1994). An outbreak of diarrhoea associated with rotavirus serotype 1 in a day care nursery in Rio de Janeiro, Brazil.. Mem Inst Oswaldo Cruz.

[21] Gallimore CI, Barreiros MAB, Brown DWG, Nascimento JP, Leite JPG (2004). Noroviruses associated with acute gastroenteritis in a children's day care facility in Rio de Janeiro, Brazil.. Braz J Med Biol Res.

[22] Morillo SG, Luchs A, Cilli A, Costa FF, Carmona RCC (2010). Characterization of rotavirus strains from day care centers: pre- and post-rotavirus vaccine era.. J Pediatr.

[23] Boom R, Sol CJ, Salimans MM, Jansen CL, Wertheim-van Dillen PM (1990). Rapid and simple method for purification of nucleic acids.. J Clin Microbiol.

[24] Leite JPG, Alfieri AA, Woods PA, Glass RI, Gentsch JR (1996). Rotavirus G and P types circulating in Brazil: characterization by RT-PCR, probe hybridization, and sequence analysis.. Arch Virol.

[25] Pereira HG, Azeredo RS, Leite JP, Candeias JA, Racz ML (1983). Electrophoretic study of the genome of human rotaviruses from Rio de Janeiro, Sao Paulo and Para, Brazil.. J Hyg (Lond).

[26] Das BK, Gentsch JR, Cicirello HG, Woods PA, Gupta A (1994). Characterization of rotavirus strains from newborns in New Delhi, India.. J Clin Microbiol.

[27] Gentsch JR, Glass RI, Woods P, Gouvea V, Gorziglia M (1992). Identification of group A rotavirus gene 4 types by polymerase chain reaction.. J Clin Microbiol.

[28] Pang XL, Preiksaitis JK, Lee B (2005). Multiplex real time RT-PCR for the detection and quantitation of norovirus genogroups I and II in patients with acute gastroenteritis.. J Clin Virol.

[29] Beuret C, Kohler D, Baumgartner A, Luthi TM (2002). Norwalk-like virus sequences in mineral waters: one-year monitoring of three brands.. Appl Environ Microbiol.

[30] Kojima S, Kageyama T, Fukushi S, Hoshino FB, Shinohara M (2002). Genogroup-specific PCR primers for detection of Norwalk-like viruses.. J Virol Methods.

[31] Vinjé J, Hamidjaja RA, Sobsey MD (2004). Development and application of a capsid VP1 (region D) based reverse transcription PCR assay for genotyping of genogroup I and II noroviruses.. J Virol Methods.

[32] Le Cann P, Ranarijaona S, Monpoeho S, Le Guyader F, Ferré V (2004). Quantification of human astroviruses in sewage using real-time RT-PCR.. Research Microbiol.

[33] Noel JS, Lee TW, Kurtz JB, Glass RI, Monroe SS (1995). Typing of human astroviruses from clinical isolates by enzyme immunoassay and nucleotide sequencing.. J Clin Microbiol.

[34] Otto TD, Vasconcellos EA, Gomes LH, Moreira AS, Degrave WM (2008). ChromaPipe: a pipeline for analysis, quality control and management for a DNA sequencing facility.. Genet Mol Res.

[35] Hall TA (1999). BioEdit: a user-friendly biological sequence alignment editor and analysis program for Windows 95/98/NT.. Nucl Acids Symp Ser.

[36] Tamura K, Dudley J, Nei M, Kumar S (2007). MEGA4: Molecular Evolutionary Genetics Analysis (MEGA) Software Version 4.0.. Mol Biol Evol.

[37] Cunliffe NA, Dove W, Gondwe JS, Thindwa BD, Greensill J (2002). Detection and characterization of human astroviruses in children with acutegastroenteritis in Blantyre, Malawi.. J Med Virol.

[38] Oh DY, Gaedicke G, Schreier E (2003). Viral agents of acute gastroenteritis in German children: Prevalence and molecular diversity.. J Med Virol.

[39] Giordano MO, Martinez LC, Isa MB, Paez RM, Nates SV (2004). Childhood astrovirus-associated diarrhea in the ambulatory setting in a Public Hospital in Cordoba city, Argentina.. Rev Inst Med Trop de Sao Paulo.

[40] CDC (2011). Updated norovirus outbreak management and disease prevention guidelines.. Morb Mortal Wkly Rep.

[41] Carvalho-Costa FA, Volotao EM, Assis RMS, Fialho AM, Andrade JSR (2011). Laboratory-based Rotavirus Surveillance During the Introduction of a Vaccination Program, Brazil, 2005–2009.. Pediatr Infect Dis J.

[42] Safadi MA, Berezin EN, Munford V, Almeida FJ, de Moraes JC (2010). Hospital-based surveillance to evaluate the impact of rotavirus vaccination in Sao Paulo, Brazil.. Pediatr Infect Dis J.

[43] Rosenfeldt V, Vesikari T, Pang XL, Zeng SQ, Tvede M (2005). Viral etiology and incidence of acute gastroenteritis in young children attending day-care centers.. Pediatr Infect Dis J.

[44] Goodgame R (2006). Norovirus gastroenteritis.. Curr Gastroenterol Rep.

[45] Tran A, Talmud D, Lejeune BT, Jovenin N, Renois F (2010). Prevalence of Rotavirus, Adenovirus, Norovirus and Astrovirus Infections and Co-infections Among Hospitalized Children in Northern France.. J Clin Microbiol.

[46] Atmar RL (2010). Noroviruses - State of the Art.. Food Environ Virol.

[47] Ribeiro LR, Giuberti RSO, Barreira DMPG, Saick KW, Leite JPG (2008). Hospitalization due to norovirus and genotypes of rotavirus in pediatric patients, state of Espirito Santo.. Mem Inst Oswaldo Cruz.

[48] Victoria M, Carvalho-Costa FA, Heinemann MB, Leite JP, Miagostovich M (2007). Prevalence and molecular epidemiology of noroviruses in hospitalized children with acute gastroenteritis in Rio de Janeiro, Brazil, 2004.. Pediatr Infect Dis J.

[49] Kale PL, Andreozzi VL, Nobre FF (2004). Time series analysis of deaths due to diarrhoea in children in Rio de Janeiro, Brazil, 1980–1998.. J Health Popul Nutr.

[50] Carvalho-Costa FA, Araujo IT, Santos de Assis RM, Fialho AM, de Assis Martins CM (2010). Rotavirus genotype distribution after vaccine introduction, Rio de Janeiro, Brazil.. Emerg Infect Dis.

[51] Cilli A, Luchs A, Morillo SG, Costa FF, Carmona RCC (2011). Characterization of rotavirus and norovirus strains: a 6-year study (2004–2009).. J Pediatr.

[52] Guix S, Caballero S, Villena C, Bartolome R, Latorre C (2002). Molecular Epidemiology of Astrovirus Infection in Barcelona, Spain.. J Clin Microbiol.

[53] Victoria M, Carvalho-Costa FA, Heinemann MB, Leite JPG, Miagostovich MP (2007). Genotypes and molecular epidemiology of human astroviruses in hospitalized children with acute gastroenteritis in Rio de Janeiro, Brazil.. J Med Virol.

[54] Fioretti J, Ferreira M, Victoria M, Vieira C, Xavier M (2011). Genetic diversity of noroviruses in Brazil..

[55] Fukuda S, Takao S, Shigemoto N, Tanizawa Y, Seno M (2010). Transition of genotypes associated with norovirus gastroenteritis outbreaks in a limited area of Japan, Hiroshima Prefecture, during eight epidemic seasons.. Arch Virol.

[56] Vega E, Vinje J (2010). Novel GII.12 norovirus strain, United States, 2009–2010.. Emerg Infect Dis.

[57] Tamura T, Nishikawa M, Anh DD, Suzuki H (2010). Molecular epidemiological study of rotavirus and norovirus infections among children with acute gastroenteritis in Nha Trang, Vietnam, December 2005–June 2006.. Jpn J Infect Dis.

[58] Buesa J, Montava R, Abu-Mallouh R, Fos M, Ribes JM (2008). Sequential evolution of genotype GII.4 norovirus variants causing gastroenteritis outbreaks from 2001 to 2006 in Eastern Spain.. J Med Virol.

[59] Siebenga J, Kroneman A, Vennema H, Duizer E, Koopmans M (2008). Food-borne viruses in Europe network report: the norovirus GII.4 2006b (for US named Minerva-like, for Japan Kobe034-like, for UK V6) variant now dominant in early seasonal surveillance.. Euro Surveill.

[60] Siebenga JJ, Vennema H, Zheng DP, Vinje J, Lee BE (2009). Norovirus illness is a global problem: emergence and spread of norovirus GII.4 variants, 2001–2007.. J Infect Dis.

[61] Castilho JG, Munford V, Resque HR, Fagundes-Neto U, Vinje J (2006). Genetic Diversity of Norovirus among Children with Gastroenteritis in Sao Paulo State, Brazil.. J Clin Microbiol.

[62] Barreira DMPG, Ferreira MSR, Fumian TM, Checon R, de Sadovsky ADI (2010). Viral load and genotypes of noroviruses in symptomatic and asymptomatic children in Southeastern Brazil.. J Clin Virol.

[63] Shinkawa N, Noda M, Yoshizumi S, Tokutake Y, Shiraishi T (2008). Molecular Epidemiology of Noroviruses Detected in Food Handler-Associated Outbreaks of Gastroenteritis in Japan.. Intervirology.

[64] Bucardo F, Nordgren J, Carlsson B, Paniagua M, Lindgren PE (2008). Pediatric norovirus diarrhea in Nicaragua.. J Clin Microbiol.

[65] Ferreira MSR, Victoria M, Carvalho-Costa FA, Vieira CB, Xavier MPTP (2010). Surveillance of norovirus infections in the state of Rio De Janeiro, Brazil 2005–2008.. J Med Virol.

[66] Pang XL, Honma S, Nakata S, Vesikari T (2000). Human Caliciviruses in Acute Gastroenteritis of Young Children in the Community.. J Infec Dis.

[67] Phillips G, Tam CC, Rodrigues LC, Lopman B (2010). Risk factors for symptomatic and asymptomatic norovirus infection in the community.. Epidemiol Infect.

